# Propranolol monotherapy versus combined propranolol-gabapentin for prevention of paroxysmal sympathetic hyperactivity after moderate-severe traumatic brain injury: a randomized controlled trial

**DOI:** 10.1186/s12871-026-03802-2

**Published:** 2026-05-08

**Authors:** Essamedin M. Negm, Ahmed M. Gouda, Mohammed El Mowafy Khatab, Essam Mohamed Elsayed Youssef, AnaSimon Alfred Foad Eskandr, Ola Mohammed Fathi, Heba Mohammed Fathi

**Affiliations:** 1 Department of Anaesthesia, ICU and Pain Management, Faculty of Medicine, Zagazig University, Zagazig, Egypt; 2 Department of Pharmacy Practice, Faculty of Pharmacy, Zagazig University, Zagazig, Egypt; 3 Department of Neurosurgery, Faculty of Medicine, Zagazig University, Zagazig, Egypt; 4 Department of Critical Care Medicine, Zagazig Chest Hospital, Zagazig, Egypt; 5 Drug Information Center, Zagazig University Hospitals, Zagazig University, Zagazig, Egypt

**Keywords:** Traumatic Brain Injuries, Propranolol, Gabapentin, Paroxysmal Sympathetic Hyperactivity, Autonomic Nervous System Diseases, Secondary Prevention, Adrenergic beta-Antagonists, Critical Care Outcomes, Length of Stay, Randomized Controlled Trial

## Abstract

**Background:**

Paroxysmal sympathetic hyperactivity (PSH) is a serious complication of traumatic brain injury (TBI), characterized by episodic hypertension (HTN), tachycardia, hyperthermia, hyperhidrosis, and dystonia. It is associated with prolonged mechanical ventilation (MV), extended ICU and hospital stays, and worse outcomes. Current guidelines lack prophylactic recommendations.

**Methodology:**

This single-center randomized controlled trial (NCT05427474) enrolled 90 adults with moderate-to-severe TBI glasgow coma scale (GCS 3–12). Participants were randomized to: standard care (Group I, *n* = 30); standard care plus propranolol (40 mg/12 h, Group II, *n* = 30); or standard care plus propranolol (40 mg/12 h) and gabapentin (100 mg/8 h, Group III, *n* = 30). The primary endpoint was PSH incidence. Secondary endpoints included ventilator days, ICU and hospital length of stay (LOS), and mortality.

**Results:**

PSH incidence was lowest in Group III (10%) vs. Group II (33.3%) and Group I (60%) (*p* < 0.001). Group II showed the shortest MV duration (5.92 ± 5.15 days) vs. Group III (9.42 ± 6.99 days, *p* = 0.047) and Group I (12.92 ± 5.98 days, *p* < 0.001). ICU LOS was shortest in Group II (9.6 ± 5.32 days) vs. Group III (14.69 ± 8.35 days, *p* = 0.017) and Group I (19.5 ± 8.19 days, *p* < 0.001). Mortality and GCS improvement did not differ significantly (*p* > 0.05).

**Conclusion:**

Prophylactic propranolol significantly reduces PSH incidence, shortens MV duration, and decreases ICU stay in moderate-to-severe TBI. Although adding gabapentin further reduces PSH, it prolongs recovery time, suggesting a trade-off between efficacy and sedative effects. These findings suggest that propranolol monotherapy is a promising prophylactic strategy, with gabapentin potentially reserved for refractory cases. However, given the study's limitations, these results should be considered hypothesis-generating and warrant confirmation in larger, multicenter trials. Mortality and neurological outcomes were comparable across groups.

**Trial registration:**

The trial was prospectively registered at ClinicalTrials.gov (NCT05427474) on June 22, 2022.

**Supplementary Information:**

The online version contains supplementary material available at 10.1186/s12871-026-03802-2.

## Introduction

Paroxysmal sympathetic hyperactivity (PSH) is a serious and frequently overlooked complication of traumatic brain injury (TBI), with its highest incidence observed in the neurocritical care population following moderate-to-severe TBI, where it is reported to affect between 8% and 33% of individuals, and up to 50% in cohorts with the most severe injuries [1–3]. It presents as recurrent surges of sympathetic activity manifesting with hypertension (HTN), tachycardia, hyperthermia, hyperhidrosis, and dystonia driven by excessive catecholamine release [4]. Beyond its distressing clinical features, PSH is an independent predictor of prolonged mechanical ventilation (MV), extended intensive care unit (ICU) and hospital stays, and substantial increases in healthcare costs [5]. Crucially, each episode can aggravate secondary brain injury through hypermetabolism, excitotoxic neuronal damage, and elevated intracranial pressure (ICP) [2], thereby fueling a broader injury cascade linked to poor outcomes in severe TBI [6]. Despite this significant clinical and economic burden, PSH prevention remains absent from current TBI management guidelines [7]. Present-day strategies are almost exclusively reactive, aiming to control symptoms after onset and often relying on anecdotal or low-quality evidence. International consensus recognizes β-blockers, such as propranolol, and gamma-aminobutyric acid (GABA) analogs, such as gabapentin, as first-line agents for PSH management [8]. However, two fundamental questions remain unanswered: first, can early administration of these agents prevent the development of PSH? Second, does combining a peripheral β-adrenergic blocker with a central GABAergic modulator offer a synergistic advantage over monotherapy [8]?

The biological rationale for such a dual prophylactic approach is compelling. Propranolol counteracts peripheral catecholamine toxicity and reduces cerebral hyperemia, while gabapentin dampens excitatory neurotransmission in thalamocortical circuits implicated in PSH pathogenesis [9]. Together, they may interrupt the autonomic dysregulation that triggers sympathetic storms. Yet, the sedative potential of gabapentin raises concerns about slower recovery trajectories highlighting a possible trade-off between maximal PSH prevention and rehabilitation speed [10].

To address these uncertainties, we conducted a randomized controlled trial designed to evaluate whether early prophylaxis with propranolol–gabapentin reduces PSH incidence compared with propranolol monotherapy or standard care. The study also assessed the impact of these interventions on recovery kinetics, measured by MV duration and ICU and hospital length of stay (LOS**)**, as well as safety outcomes including mortality and neurological status.

## Patients and methods

### Study design and setting

This prospective randomized controlled trial was conducted in the ICU at Zagazig University Hospitals over an 11-month period, from December 1, 2022, to October 31, 2023. The study was designed and reported in accordance with the Consolidated Standards of Reporting Trials (CONSORT) guidelines. A total of 90 patients with moderate-to-severe TBI admitted to the ICU during this period were enrolled.

### Ethical approval

The study protocol was approved by the Institutional Review Board (IRB) of the Faculty of Medicine, Zagazig University (IRB No. 9076/8-11-2021). Written informed consent was obtained from the first-degree relatives of all participants after a detailed explanation of the study.

### Inclusion criteria

Patients were eligible if they were admitted to the ICU with moderate to severe TBI, defined by Glasgow Coma Scale (GCS) score of 9–12 for moderate cases and 3–8 for severe cases [11]. Additional eligibility requirements included an age between 18 and 64 years, both male and female patients, and the availability of a first-degree relative able to provide written informed consent.

### Exclusion criteria

Patients were excluded if they had pre-existing brain dysfunction, allergy to propranolol or gabapentin, significant cardiac disease, chronic kidney disease, cervical spine injury, or chronic obstructive pulmonary disease (COPD). Patients presenting with bradycardia, uncontrolled HTN, or hypotension upon ICU admission were also excluded. Those receiving active propranolol or gabapentin therapy at screening were excluded.

### Sample size calculation

The sample size was calculated a priori based on the primary outcome (PSH incidence). Assuming a control incidence of 50% and a reduction to 30% with active prophylaxis, with α = 0.05 and 80% power, the required sample size was 81 patients. To account for potential dropouts, the sample size was increased to 90 patients (30 per group). The calculation was performed using G*Power software (version 3.1.9.4).

### Method of randomization

After obtaining informed consent, patients were randomly allocated in a 1:1:1 ratio into three groups using a computer-generated sequence prepared by an independent investigator. Allocation was concealed using sequentially numbered, sealed, opaque envelopes opened after enrollment.

### Blinding of the study

The study medications (propranolol, gabapentin, and their respective placebos) were prepared in identical capsules and packaged in neutral, coded containers by a pharmacist not involved in patient care or data analysis. Standard care was provided uniformly across all groups, with placebo capsules administered at the same schedule as the active drugs to maintain treatment masking. Treating ICU physicians were aware of group allocation due to the need for dose adjustment and safety monitoring, but they did not participate in outcome assessments. The clinical team responsible for diagnosing PSH, recording ventilator duration, ICU/hospital LOS, and compiling outcome data was blinded to group assignments. Importantly, this clinical endpoint adjudication team was responsible for diagnosing PSH strictly according to the international consensus criteria [12] and operated independently from the treating physicians. Data analysts were also blinded until after the completion of statistical analysis.

### Study groups

Patients were randomized into three groups (1:1:1): Control (standard TBI care), Propranolol (standard care plus propranolol), and Combination (standard care plus propranolol and gabapentin), administered orally or via nasogastric tube (PO/NGT). Drug dosing and mechanisms are detailed in Supplementary Table 1. Treatment continued until discharge or death, and all other ICU care followed institutional TBI protocols.

### Baseline assessment

Upon ICU admission, all patients underwent a comprehensive baseline evaluation, including detailed medical history (age, sex, comorbidities, surgical history, current medications, and final diagnosis), full clinical examination, and routine laboratory investigations. Primary and secondary trauma surveys were performed for all patients. A small number of patients had a remote history of gabapentin use that had been discontinued prior to admission; these patients were not on active therapy at the time of injury and were managed per protocol as gabapentin-naïve.

### Interventions

All patients received standard TBI management according to the institutional ICU protocol [13]. This included ICP monitoring in patients with GCS ≤ 8, maintenance of mean arterial pressure (MAP) above 80 mmHg, MV for patients with PaO₂/FiO₂ < 300, and initiation of enteral nutrition within 48 h of admission. For Groups II and III, trial medications were initiated within 6 h of ICU admission and continued until hospital discharge or death. Group II received propranolol only, while Group III received both propranolol and gabapentin. The dosing regimens were propranolol 40 mg every 12 h and gabapentin 100 mg every 8 h. These doses were selected based on previously published clinical series and with a primary focus on safety for prophylactic use in critically ill patients.

### Study flow

The sequence of data presentation in the Results section was predefined to follow the clinical trajectory of patients. Study flow included: baseline characteristics → trauma severity → initial clinical and radiological assessment → ICU admission and follow-up → primary outcome (PSH incidence and profile) → ICU complications and management → duration of MV and ICU stay → discharge status → neurological recovery.

### Study outcomes

#### Primary outcome

The primary outcome was PSH incidence, diagnosed according to international consensus criteria [12]. Episodes were defined by the presence of at least three simultaneous features per day: tachycardia (heart rate (HR) > 120 bpm), HTN (systolic blood pressure (SBP) > 140 mmHg), tachypnea (respiratory rate (RR) > 30 breaths/min), diaphoresis, with or without dystonia or hyperthermia (> 38.5 °C), in the absence of other causes.

#### Secondary outcomes

Secondary outcomes were selected to measure the potential clinical and resource utilization benefits stemming from the prevention of PSH. These included: (1) duration of MV, as PSH episodes directly impede weaning; (2) ICU and hospital LOS, as PSH prolongs acute care; (3) all-cause mortality; (4) change in GCS score from admission to discharge, as a measure of neurological recovery; and (5) ICU complications. Infectious complications (e.g., pneumonia, sepsis) were identified and monitored from ICU admission until hospital discharge using standard clinical and microbiological criteria as part of the safety assessment.

### Statistical analysis

Data were analyzed using IBM SPSS Statistics (version 23.0). Continuous variables are presented as mean ± standard deviation (SD), and categorical variables as frequencies and percentages (n, %). The primary outcome (PSH incidence) was compared across groups using the Chi-square test, with pairwise comparisons using Bonferroni correction. Results are reported as counts (%) with relative risk and 95% confidence intervals (CIs). For secondary continuous outcomes (MV duration, ICU LOS, hospital LOS), between-group comparisons were performed using one-way analysis of variance (ANOVA). Post-hoc pairwise comparisons were conducted using Tukey’s Honestly Significant Difference (HSD) test. When ANOVA assumptions were violated, the non-parametric Kruskal-Wallis H test was employed, followed by Dunn’s test with Bonferroni adjustment. Within-group changes in GCS were analyzed using paired t-test. A post-hoc descriptive analysis of daily HR and SBP was performed over 14 days, with results presented as median and interquartile range (IQR). All analyses were conducted on an intention-to-treat (ITT) basis. Durations for patients who died were censored at death. Treatment administration was monitored via electronic records; adherence was high (~ 92% of doses administered), with minor deviations due to transient clinical circumstances. A two-tailed p-value < 0.05 was considered statistically significant.

## Results

### Study population and baseline characteristics

**Fig. 1:**
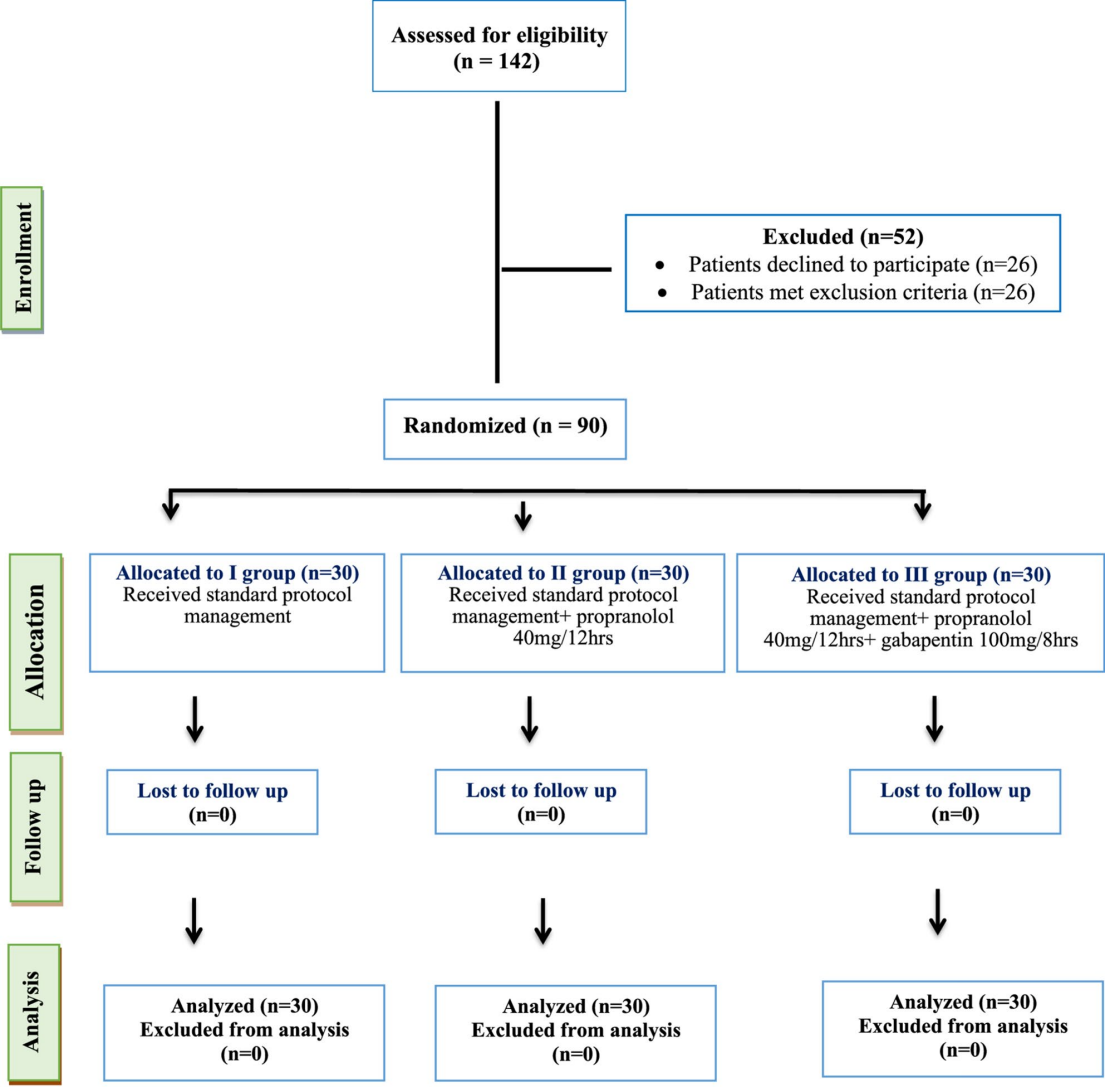
Consort flow diagram of patient population through each stage of the randomized trial

Of 142 patients assessed for eligibility, 52 were excluded (26 declined, 26 met exclusion criteria). Ninety patients were randomized equally into three groups (*n* = 30 each) and completed the study with no loss to follow-up (Fig. 1). The three groups were well matched for demographic, clinical, and injury characteristics, with no statistically significant differences (Table 1). Age, sex, body mass index (BMI), comorbidities including HTN, diabetes mellitus (DM), and hepatitis C virus (HCV) infection, and medication history were comparable across groups. Trauma mechanism and TBI severity were similar across groups. Road traffic accidents (RTA) accounted for > 90% of injuries, and severe TBI predominated (60–63%) (Table 2). Baseline computed tomography (CT) findings were comparable, with tense cerebral edema as the most common finding (53–57%). Extracranial injuries and laboratory parameters showed no significant intergroup differences (Table 3).

**Table 1:** Demographic, medical, surgical, and medication history of the three studied groups

Variable	Group I (*n* = 30)	Group II (*n* = 30)	Group III (*n* = 30)	Test value	*P* value	Sig.
Age (years)	31.03 ± 10.97	32.27 ± 12.20	31.40 ± 11.28	0.091	0.913	NS
Range	18–64	18–62	18–60			
Gender				0.934	0.627	NS
Male	24 (80.0%)	22 (73.3%)	25 (83.3%)			
Female	6 (20.0%)	8 (26.7%)	5 (16.7%)			
BMI (kg/m²)	24.07 ± 4.53	24.03 ± 4.14	23.63 ± 3.32	0.109	0.897	NS
Range	18.5–33.5	18.5–30.3	18.9–30.2			
Past medical & surgical history				0.271	0.873	NS
Absent	18 (60.0%)	16 (53.3%)	17 (56.7%)			
Present	12 (40.0%)	14 (46.7%)	13 (43.3%)			
Smoking				0.117	0.943	NS
Non-smoker	23 (76.7%)	22 (73.3%)	22 (73.3%)			
Current smoker	7 (23.3%)	8 (26.7%)	8 (26.7%)			
Comorbidities						
DM	4 (13.3%)	4 (13.3%)	4 (13.3%)	0.0	0.999	NS
HTN	4 (13.3%)	5 (16.7%)	5 (16.7%)	0.169	0.919	NS
HCV	0 (0.0%)	4 (13.3%)	1 (3.3%)	5.506	0.064	NS
Previous surgeries						
Thyroidectomy	1 (3.3%)	0 (0.0%)	0 (0.0%)	2.022	0.364	NS
Appendectomy	1 (3.3%)	1 (3.3%)	3 (10.0%)	1.694	0.429	NS
Cholecystectomy	0 (0.0%)	1 (3.3%)	0 (0.0%)	2.022	0.364	NS
Past medication history				0.0	0.999	NS
Present	9 (30.0%)	9 (30.0%)	9 (30.0%)			
L-thyroxin	1 (3.3%)	0 (0.0%)	0 (0.0%)	2.022	0.364	NS
Insulin	0 (0.0%)	2 (6.7%)	1 (3.3%)	2.069	0.355	NS
OHD	4 (13.3%)	2 (6.7%)	3 (10.0%)	0.741	0.690	NS
Anti-HTN	4 (13.3%)	5 (16.7%)	5 (16.7%)	0.169	0.919	NS

Data are presented as mean ± SD or number (%). *NS* Non-significant (*p* > 0.05), *BMI* Body Mass Index, *HTN* Hypertension, *DM* Diabetes Mellitus, *HCV* Hepatitis C Virus, *OHD* Oral hypoglycemic drugs, *Anti-HTN* Antihypertensive drugs

**Table 2:** Comparison between the three groups studied regarding type of trauma and level of TBI

Variable	**Group I (n = 30)**	**Group II (n = 30)**	**Group III (n = 30)**	Test value	*p*-value	Sig.
Type of trauma						
Direct trauma	0 (0.0%)	1 (3.3%)	2 (6.7%)	2.596	0.627	NS
FFH	1 (3.3%)	2 (6.7%)	1 (3.3%)			
RTA	29 (96.7%)	27 (90.0%)	27 (90.0%)			
Level of TBI						
Moderate	11 (36.7%)	11 (36.7%)	12 (40.0%)	0.095	0.954	NS
Severe	19 (63.3%)	19 (63.3%)	18 (60.0%)			

Data are presented as number (%). *NS* Non-significant (*p* > 0.05), *FFH* Falling from height, *RTA* Road traffic accident, *TBI* Traumatic brain injury

**Table 3:** Comparison between the three groups studied regarding radiological, clinical, and laboratory characteristics at admission

Parameter	Group I (*n* = 30)	Group II (*n* = 30)	Group III (*n* = 30)	Test value	*P* value	Sig.
CT Brain						
Tense cerebral edema	16 (53.3%)	17 (56.7%)	16 (53.3%)	0.090	0.956	NS
Brain contusion	6 (20.0%)	6 (20.0%)	7 (23.3%)	0.133	0.935	NS
Intracranial hemorrhage	6 (20.0%)	8 (26.7%)	9 (30.0%)	0.818	0.664	NS
SAH	10 (33.3%)	8 (26.7%)	6 (20.0%)	1.364	0.506	NS
EDH	1 (3.3%)	2 (6.7%)	3 (10.0%)	1.071	0.585	NS
SDH	6 (20.0%)	1 (3.3%)	5 (16.7%)	4.038	0.133	NS
DAI	0 (0%)	1 (3.3%)	0 (0%)	2.022	0.364	NS
CT Chest/PAUS/Orthopedic						
Lung contusion	10 (33.3%)	13 (43.3%)	11 (36.7%)	0.662	0.718	NS
Rib fracture	0 (0%)	0 (0%)	1 (3.3%)	2.022	0.364	NS
Thoracic spine injury	0 (0%)	1 (3.3%)	0 (0%)	2.022	0.364	NS
Lumbar spine injury	1 (3.3%)	0 (0%)	0 (0%)	2.022	0.364	NS
Splenic injury	1 (3.3%)	1 (3.3%)	1 (3.3%)	0.000	1.000	NS
Hepatic injury	1 (3.3%)	0 (0%)	1 (3.3%)	1.023	0.600	NS
Pelvic fracture	1 (3.3%)	2 (6.7%)	1 (3.3%)	0.523	0.770	NS
Clinical data						
GCS (Mean ± SD)	7.90 ± 2.76	8.07 ± 2.41	8.03 ± 2.30	0.037	0.963	NS
HR (Mean ± SD)	92.93 ± 8.90	91.27 ± 10.24	92.67 ± 9.00	0.272	0.762	NS
SBP (Mean ± SD)	119.67 ± 11.44	120.67 ± 9.17	122.17 ± 8.68	0.491	0.614	NS
DBP (Mean ± SD)	75.33 ± 8.60	75.67 ± 9.71	77.00 ± 6.51	0.332	0.718	NS
O₂ saturation (%)	96.13 ± 1.80	95.33 ± 2.15	95.40 ± 2.21	1.392	0.254	NS
Hb (g/dl)	12.73 ± 1.56	12.59 ± 1.77	12.54 ± 1.15	0.135	0.874	NS
PTT (sec)	28.47 ± 3.01	29.70 ± 3.51	29.57 ± 3.79	1.153	0.320	NS
INR	1.08 ± 0.08	1.07 ± 0.08	1.07 ± 0.10	0.085	0.919	NS
Characteristic labs						
Leukocytosis	4 (13.3%)	6 (20.0%)	5 (16.7%)	0.480	0.787	NS
CRP increased	4 (13.3%)	6 (20.0%)	5 (16.7%)	0.480	0.787	NS
Liver enzymes ↑	3 (10.0%)	2 (6.7%)	2 (6.7%)	0.310	0.856	NS
Sputum culture +ve	4 (13.3%)	4 (13.3%)	4 (13.3%)	0.000	1.000	NS
Blood culture +ve	0 (0%)	2 (6.7%)	0 (0%)	4.091	0.129	NS
Urine culture +ve	0 (0%)	1 (3.3%)	0 (0%)	2.022	0.364	NS
CVL culture +ve	0 (0%)	2 (6.7%)	2 (6.7%)	2.093	0.351	NS

Data are presented as mean ± SD or number (%). *NS* Non-significant (*p* > 0.05), *GCS* Glasgow Coma Scale, *HR* Heart rate, *SBP* Systolic blood pressure, *DBP* Diastolic blood pressure, *Hb* Hemoglobin, *PAUS* Pelvic-abdominal ultrasound, *PTT* Partial thromboplastin time, *INR* International normalized ratio, *CRP* C-reactive protein, *SAH* Subarachnoid hemorrhage, *EDH* Epidural hematoma, *SDH* Subdural hemorrhage, *DAI* Diffuse axonal injury, *CVL* Central venous line

All patients were admitted for management of moderate-to-severe TBI. Secondary admission triggers (e.g., disturbed consciousness, pneumonia, respiratory distress (RD)) occurred sporadically with no systematic group distribution (all *p* > 0.05) (Table 4). On follow-up imaging, persistent brain edema was significantly less frequent in the treatment groups, particularly Group III (3.3% vs. 26.7% in controls, *p* = 0.020). Other radiological findings did not differ significantly.

**Table 4:** Comparison between the three studied groups regarding causes of ICU admission and follow-up radiological/clinical findings.=

Variable	Group I (*n* = 30)	Group II (*n* = 30)	Group III (*n* = 30)	Test value*	*p*-value	Sig.
Cause of ICU admission						
DCL	2 (6.7%)	2 (6.7%)	1 (3.3%)	0.424	0.809	NS
Pneumonia	1 (3.3%)	1 (3.3%)	1 (3.3%)	0.000	1.000	NS
RD	0 (0%)	0 (0%)	2 (6.7%)	4.091	0.129	NS
Lung collapse	1 (3.3%)	0 (0%)	1 (3.3%)	1.023	0.600	NS
Pneumothorax	1 (3.3%)	0 (0%)	1 (3.3%)	1.023	0.600	NS
Fits	1 (3.3%)	1 (3.3%)	2 (6.7%)	0.523	0.770	NS
Hematemesis	0 (0%)	1 (3.3%)	0 (0%)	2.022	0.364	NS
Postoperative	1 (3.3%)	2 (6.7%)	2 (6.7%)	0.424	0.809	NS
Follow-up CT brain						
Brain edema (resolving / mild / moderate / tense)	11 / 2 / 9 / 8	10 / 3 / 11 / 6	8 / 11 / 10 / 1	15.008	0.020	S
Brain contusion (absent / resolving / present)	25 / 1 / 4	25 / 1 / 4	26 / 1 / 3	0.208	0.995	NS
Epidural rim (absent / present)	29 / 1	30 / 0	28 / 2	2.069	0.355	NS
Intracranial hematoma (absent / resolving / present)	24 / 2 / 4	22 / 1 / 7	21 / 4 / 5	3.084	0.544	NS
SAH (absent / resolving / present)	20 / 4 / 6	22 / 6 / 2	23 / 5 / 2	3.815	0.432	NS
SDH (absent / present)	26 / 4	28 / 2	25 / 5	1.450	0.484	NS
EDH (absent / resolving / present)	29 / 0 / 1	28 / 2 / 0	27 / 3 / 0	4.871	0.301	NS
DAI (absent / present)	27 / 3	28 / 2	28 / 2	0.310	0.856	NS
Skull fracture (absent / depressed / fissure / base)	25 / 4 / 0 / 1	24 / 3 / 2 / 1	25 / 4 / 1 / 0	3.209	0.782	NS
Follow-up chest & abdominal						
Lung contusion (absent / resolving / present)	21 / 3 / 6	22 / 1 / 7	27 / 0 / 3	6.011	0.198	NS
Intraperitoneal free fluid (absent / minimal)	28 / 2	26 / 4	28 / 2	1.098	0.578	NS
Spleen (absent / injury / splenectomy)	29 / 1 / 0	29 / 0 / 1	29 / 1 / 0	3.000	0.558	NS
Hepatic injury (absent / present)	29 / 1	30 / 0	28 / 2	2.069	0.355	NS
Rib fracture (absent / present)	30 / 0	30 / 0	29 / 1	2.022	0.364	NS
Operation (absent / cranial / non-cranial / both)	17 / 10 / 3 / 0	19 / 7 / 3 / 1	14 / 15 / 1 / 0	6.965	0.324	NS

Data are presented as number (%). *NS* Non-significant (*p* > 0.05), *S* Significant (*p* < 0.05), *DCL* Disturbed conscious level, *RD* Respiratory distress, *CT* Computed tomography, *PAUS* Pelvic-abdominal ultrasound, *SAH* Subarachnoid hemorrhage, *SDH* Subdural hemorrhage, *EDH* Epidural hematoma, *DAI* Diffuse axonal injury, *ICU* Intensive care unit

### Primary outcome: PSH incidence and symptom profile

PSH incidence differed significantly across groups (*p* < 0.001). It occurred in 60% of Group I (control), 33.3% of Group II (propranolol), and 10% of Group III (combination). Pairwise comparisons were all significant (Table 5).

**Table 5:** Comparison between the three studied groups regarding PSH incidence

PSH	Group I (*n* = 30)	Group II (*n* = 30)		Group III (*n* = 30)	Test value*		*p*-value	Sig.
Absent	12 (40.0%)	20 (66.7%)		27 (90.0%)	16.632		0.000	HS
Present	18 (60.0%)	10 (33.3%)		3 (10.0%)				

**Post Hoc analysis**								
**Group I Vs Group II**			**Group I Vs Group III**			**Group II Vs Group III**		
0.038			0.000			0.028		

Data are presented as number (percentage). Test value*: Chi-square test was used. Post hoc analysis was performed to detect intergroup differences. *p* < 0.05 is considered significant (S); *p* < 0.01 is considered highly significant (HS); *NS* not significant.* PSH* Paroxysmal Sympathetic Hyperactivity

Mean time to PSH onset was similar across groups (7.3–9.1 days, *p* = 0.082). Regarding symptom distribution, significant intergroup differences were observed for tachycardia (*p* = 0.012), HTN (*p* = 0.002), and sweating (*p* = 0.012), with the lowest rates in Group II and Group III (Table 6). Tachypnea, posturing, and hyperthermia did not differ significantly. Use of rescue medications mirrored PSH incidence (60% in Group I, 33.3% in Group II, 10% in Group III). Both treatment groups showed sustained attenuation of daily HR and SBP compared to controls, with Group III exhibiting the most stable reduction (Supplementary Table 6).

**Table 6:** Comparison between the three studied groups regarding PSH onset and symptoms

Parameter	***Group I (n> = 30)***	***Group II (n = 30)***	***Group III (n = 30)***	Test value*	*p*-value	Sig.	Overall (n = 31*)
Onset of PSH (days)	7.27 ± 3.06 (1–14)	8.03 ± 2.97 (1–14)	9.1 ± 3.34 (2–14)	2.568	0.082	NS	—
Tachypnea	9 (50.0%)	2 (20.0%)	1 (33.3%)	2.479	0.290	NS	12 (38.7%)
Tachycardia	14 (77.8%)	2 (20.0%)	2 (66.7%)	8.914	0.012	S	18 (58.1%)
HTN	14 (77.8%)	1 (10.0%)	1 (33.3%)	12.269	0.002	HS	16 (51.6%)
Sweating	14 (77.8%)	2 (20.0%)	2 (66.7%)	8.914	0.012	S	18 (58.1%)
Posturing	6 (33.3%)	1 (10.0%)	0 (0.0%)	2.971	0.226	NS	7 (22.6%)
Hyperthermia	9 (50.0%)	3 (30.0%)	1 (33.3%)	1.157	0.561	NS	13 (41.9%)

Data are presented as mean ± SD (range) or number (percentage). ANOVA test was used for continuous variables and Chi-square test for categorical variables. *p* < 0.05 is considered significant (S); *p* < 0.01 highly significant (HS); *NS* not significant. *PSH* Paroxysmal Sympathetic Hyperactivity

*Overall descriptive data (*n* = 31 patients with PSH) are presented for symptoms

### Secondary outcomes

Overall complication rates were similar across groups (26.7%, 30%, and 16.7% in Groups I–III, respectively; *p* > 0.05). Pneumonia, ventilator-associated pneumonia (VAP), sepsis, and drug-induced hepatitis occurred at comparable frequencies. Hypotensive episodes were infrequent and predominantly sepsis-related, with no excess risk in propranolol-containing groups (Supplementary Table 2). MV requirement and sedation practices did not differ significantly across groups (Supplementary Table 3).

Significant intergroup differences were observed for MV duration, ICU stay, and hospital LOS (*p* < 0.01). Group II (propranolol) had the shortest MV duration (5.9 ± 5.2 days), ICU stay (9.6 ± 5.3 days), and hospital LOS (11.9 ± 6.1 days), followed by Group III, with Group I having the longest durations (all *p* < 0.05) (Table 7).

**Table 7:** Comparison between the three studied groups regarding Mechanical ventilation duration, ICU length of stay, and hospital length of stay (days)

Parameter	***Group I (n = 30)***	***Group II (n = 30)***	***Group III (n = 30)***	Test value	*P*-value	Sig.	Post Hoc (LSD)
MV duration (days)	12.92 ± 5.98 (1–23)	5.92 ± 5.15 (1–25)	9.42 ± 6.99 (1–37)	7.865	0.001	HS	I vs. II: 0.000** I vs. III: 0.047* II vs. III: 0.047*
ICU stay (days)	19.50 ± 8.19 (6–39)	9.60 ± 5.32 (2–25)	14.69 ± 8.35 (1–37)	10.910	< 0.001	HS	I vs. II: 0.000** I vs. III: 0.025* II vs. III: 0.017*
Hospital stay (days)	21.57 ± 9.93 (6–39)	11.87 ± 6.08 (3–27)	17.03 ± 8.59 (5–37)	10.126	< 0.001	HS	I vs. II: 0.000** I vs. III: 0.038* II vs. III: 0.019*

Data are presented as mean ± SD (range). These analyses include all randomized patients on an intention-to-treat basis, with durations for deceased patients censored at the time of death. Mortality rates by group are provided in Supplementary Table 4

*LSD* least significant difference, *NS* Non-significant (*p* > 0.05), *S* Significant (*p* < 0.05), *HS* Highly significant (*p* < 0.01). * *p* < 0.05, ** *p* < 0.01 (Post Hoc LSD test). *MV* Mechanical ventilation, *ICU* Intensive care unit

Discharge disposition and mortality did not differ significantly between groups (Supplementary Table 4). Although GCS improved significantly within each group from admission to discharge (all *p* < 0.001), intergroup differences were not significant (Supplementary Table 5).

## Discussion

This randomized controlled trial demonstrated that propranolol, alone or with gabapentin, significantly reduced PSH incidence following moderate-to-severe TBI compared with standard care alone [14]. Propranolol effectively attenuated cardiovascular manifestations such as tachycardia and HTN [15], while adding gabapentin broadened symptomatic control across the PSH spectrum [16]. Secondary outcomes showed shorter MV, ICU stay, and hospital stay in the propranolol group, though these benefits were less consistent with combination therapy. Neurological recovery (GCS) improved significantly within all groups without intergroup differences, and mortality remained comparable. The three study groups were well balanced at baseline with no significant differences across demographic, clinical, radiological, or laboratory variables (Table 1). Trauma characteristics were evenly distributed, with RTA accounting for the majority of injuries and severe TBI predominating across all groups. Follow-up imaging showed a higher frequency of persistent brain edema in the control group compared with treatment groups, which may reflect a neuroprotective effect of propranolol and possibly gabapentin through modulation of sympathetic overdrive a process involving excitotoxicity, inflammation, and metabolic dysregulation linked to poor outcomes in severe TBI [6]. Taken together, this baseline homogeneity minimizes the potential for confounding and supports the validity of attributing subsequent differences in outcomes to the pharmacological interventions under investigation [17].

PSH is a well-recognized complication of severe TBI, associated with prolonged MV, extended ICU stay, and worse neurological recovery [18]. Our study focused on PSH incidence within this high-risk population, which aligns with reported rates of 8–33% (up to 50% in severe cohorts) [1, 2], differing from etiological prevalence reviews where TBI accounts for a smaller proportion of all PSH cases [19]. Beyond diagnostic challenges, PSH has important clinical implications: it is associated with prolonged MV, extended ICU and hospital stay, and, potentially, worse neurological recovery. Accordingly, strategies to prevent or attenuate PSH are a key priority in neurocritical care research [20]. Our findings demonstrate a clear reduction in PSH with β-adrenergic blockade. Compared with standard care (60%), propranolol reduced incidence to 33.3%, and the combination further lowered it to 10% (ARR 26.7–50%; NNT 2–4). The symptom pattern aligned with expected pharmacodynamics: propranolol attenuated tachycardia and HTN [21], while gabapentin broadened control to non-cardiovascular features [7]. The significant improvement in critical care outcomes with propranolol suggests a broader therapeutic benefit beyond discrete PSH episodes, supporting early intervention in high-risk patients [22].

The apparent paradox combination therapy achieving the lowest PSH incidence yet propranolol monotherapy showing the shortest MV, ICU, and hospital stays deserves consideration. Propranolol’s superiority likely stems from targeted mitigation of catecholamine toxicity without gabapentin’s sedative burden [22]. Cardiovascular symptom control may be sufficient to accelerate recovery, while combination therapy may be reserved for refractory cases [12]. Possible explanations include gabapentin’s sedative profile modestly slowing wakefulness and extubation readiness, multifactorial determinants of length-of-stay diluting the impact of PSH prevention, and sample size limitations reducing precision around secondary endpoints. These hypotheses warrant further targeted studies [23].

Our results are broadly consistent with prior work. β-blocker use has been associated with reduced autonomic storm burden and improved outcomes [16, 24], with propranolol serving as first-line therapy in protocolized pathways [25]. Conversely, not all series demonstrate uniform benefit on length-of-stay or ventilator duration, underscoring that PSH is only one determinant of critical-care course [26]. Evidence for gabapentin remains adjunctive [27]. Our randomized data add two signals: (1) propranolol effectively prevents PSH with strong cardiovascular control, and (2) early combination maximizes PSH prevention without superior resource utilization.

Clinically, these findings support early propranolol after ICU admission with vigilant monitoring, adding gabapentin when broader PSH features emerge [15, 28]. Safety outcomes showed no excess hemodynamic risk with propranolol [29], and sedation practices were balanced across groups, though the combination group showed a trend toward higher propofol use [30, 31].

The divergence between PSH incidence and resource utilization propranolol monotherapy achieving shorter ICU stay (9.6 vs. 19.5 days) despite higher PSH incidence than combination therapy underscores the multifactorial nature of ICU outcomes [18, 32]. Propranolol may confer a more favorable balance by targeting cardiovascular surges without additional sedation [18].

These findings resonate with observational cohorts linking β-blockers to shorter ICU stay and improved survival [32, 33], though randomized data remain limited: a pilot RCT by Cruickshank et al. suggested propranolol reduced sympathetic surges and ICU instability, but sample size constraints precluded definitive conclusions regarding length-of-stay [34]. Gabapentin’s sedative profile may partly offset gains in extubation readiness. Case series (e.g., Baguley et al.) describe benefit in controlling paroxysmal sympathetic storms and neuropathic features [35], but no study has demonstrated consistent reductions in MV duration or ICU length-of-stay [20, 25]. Overall, propranolol reduced PSH and translated this into shorter MV, ICU stay, and hospitalization [36]. Combination therapy, while achieving lowest PSH incidence, did not confer parallel ICU efficiency gains [18, 20]. Propranolol emerges as the most pragmatic intervention, with combination therapy reserved for refractory cases [32]. Discharge outcomes showed favorable trends without statistical significance [36–38]. Neurological recovery (GCS) improved significantly within each group, but intergroup differences were not significant [39, 40], aligning with literature where β-blockers consistently reduce acute complications but direct neurological improvement remains less definitive [41, 42].

### Limitations

Several limitations should be acknowledged. First, the single-center design and moderate sample size (*n* = 90) may limit generalizability. Second, unmasking due to drug side effects remains possible despite blinded assessment. Third, follow-up was restricted to acute hospitalization, precluding long-term functional assessment. Fourth, competing mortality complicates LOS interpretation. Fifth, the large treatment effects (54–51% reductions) are biologically plausible but require replication. Finally, findings are specific to gabapentin-naïve patients; applicability to other populations remains unexplored.

### Future work

Future research should prioritize multicenter trials for validation, dose-optimization studies, comparative effectiveness against other sympatholytics, biomarker development for precise phenotyping, and exploration of gabapentin’s immunomodulatory properties.

## Conclusion

This trial demonstrates that early propranolol significantly reduces PSH incidence and improves critical care outcomes in moderate-to-severe TBI. While the combination showed superior PSH prevention, it conferred no added benefit for resource utilization. Propranolol represents a promising prophylactic strategy, with gabapentin reserved for refractory cases. Replication in larger trials is needed before definitive recommendations.

## Supplementary Information

Supplementary Material 1.

## Data Availability

The datasets generated and analyzed during the current study are not publicly available but are available from the corresponding author upon a reasonable request.

## Abbreviations

AKI: Acute kidney injury
ANOVA: Analysis of variance
BB: Beta blocker
BMI: Body mass index
CI: Confidence interval
CONSORT: Consolidated Standards of Reporting Trials
COPD: Chronic obstructive pulmonary disease
CRP: C-reactive protein
CT: Computed tomography
CVL: Central venous line
DAI: Diffuse axonal injury
DBP: Diastolic blood pressure
DCL: Disturbed conscious level
DM: Diabetes mellitus
EDH: Epidural hematoma
FFH: Falling from height
GABA: Gamma-aminobutyric acid
GCS: Glasgow coma scale
Hb: Hemoglobin
HCV: Hepatitis C virus
HR: Heart rate
HS: Highly significant
HSD: Honestly significant difference
HTN: Hypertension
ICP: Intracranial pressure
ICU: Intensive care unit
ITT: Intention-to-Treat
IJV: Internal jugular vein
INR: International normalized ratio
IQR: Interquartile range
IRB: Institutional review board
LOS: Length of stay
LSD: Least significant difference
MAP: Mean arterial pressure
MV: Mechanical ventilation
NGT: Nasogastric tube
NS: Non-significant
OHD: Oral hypoglycemic drug
PAUS: Pelvic-abdominal ultrasound
PO: Per os (orally)
PSH: Paroxysmal sympathetic hyperactivity
PTT: Partial thromboplastin time
RD: Respiratory distress
RR: Respiratory rate
RTA: Road traffic accident
S: Significant
SAH: Subarachnoid hemorrhage
SBP: Systolic blood pressure
SD: Standard deviation
SDH: Subdural hemorrhage
TBI: Traumatic brain injury
VAP: Ventilator-associated pneumonia
