# Supplementary material for: Propranolol monotherapy versus combined propranolol-gabapentin for prevention of paroxysmal sympathetic hyperactivity after moderate-severe traumatic brain injury: a randomized controlled trial
Source: BMC Anesthesiol. 2026 May 8;26:291. doi: 10.1186/s12871-026-03802-2 (PMC13159313; doi:10.1186/s12871-026-03802-2)
Supplement: Supplementary file 1 — Supplementary Material 1. [file 12871_2026_3802_MOESM1_ESM.docx]

**Supplementary Table (1):** Dosing Regimens for the Study Intervention Groups.

| **Drug** | **Route** | **Dose** | **Mechanism of action** | **Reference** |
| --- | --- | --- | --- | --- |
| Propranolol | PO/NGT | 40mg/12hrs | Peripheral non-selective beta blocker )BB) | [45, 46] |
| Gabapentin | PO/NGT | 100mg/8hrs | Central GABA agonist | [22] |

BB; beta blocker PO: Per Os (orally); NGT: Nasogastric Tube; GABA: γ-Aminobutyric Acid.
The referenced protocols are based on the cited literature and institutional guidelines for TBI management*.*

**Supplementary Table (2): Complications During ICU Stay and Renal Safety Outcomes**

| **Complication** | **Group I (n=30)** | **Group II (n=30)** | **Group III (n=30)** | **Test value*** | **p-value** | **Sig.** |
| --- | --- | --- | --- | --- | --- | --- |
| **Overall complications** | 8 (26.7%) | 9 (30.0%) | 5 (16.7%) | 1.564 | 0.457 | NS |
| **Pneumonia** | 2 (6.7%) | 2 (6.7%) | 1 (3.3%) | 0.424 | 0.809 | NS |
| **RD** | 1 (3.3%) | 0 (0.0%) | 0 (0.0%) | 2.022 | 0.364 | NS |
| **Pneumothorax** | 0 (0.0%) | 0 (0.0%) | 1 (3.3%) | 2.022 | 0.364 | NS |
| **Sepsis** | 3 (10.0%) | 7 (23.3%) | 4 (13.3%) | 2.199 | 0.333 | NS |
| **Drug-induced hepatitis** | 3 (10.0%) | 1 (3.3%) | 1 (3.3%) | 1.694 | 0.429 | NS |
| **VAP** | 1 (3.3%) | 1 (3.3%) | 1 (3.3%) | 0.000 | 1.000 | NS |
| **Fits** | 2 (6.7%) | 2 (6.7%) | 0 (0.0%) | 2.093 | 0.351 | NS |
| **CVL infection** | 0 (0.0%) | 0 (0.0%) | 2 (6.7%) | 4.091 | 0.129 | NS |
| **IJV infection** | 0 (0.0%) | 0 (0.0%) | 1 (3.3%) | 2.022 | 0.364 | NS |
| **Neck edema** | 0 (0.0%) | 0 (0.0%) | 1 (3.3%) | 2.022 | 0.364 | NS |
| **Pulmonary embolism** | 0 (0.0%) | 0 (0.0%) | 1 (3.3%) | 2.022 | 0.364 | NS |
| **Renal Safety Outcomes** | | | | | | |
| **Acute Kidney Injury (AKI)** | 6 (20.0%) | 5 (16.7%) | 7 (23.3%) | 0.480 | 0.787 | NS |
| **- KDIGO Stage 1** | 4 (13.3%) | 3 (10.0%) | 5 (16.7%) | - | - | - |
| **- KDIGO Stage 2** | 2 (6.7%) | 2 (6.7%) | 2 (6.7%) | - | - | - |
| **- KDIGO Stage 3** | 0 (0.0%) | 0 (0.0%) | 0 (0.0%) | - | - | - |
| **Peak Serum Creatinine (mg/dL), mean ± SD** | 1.3 ± 0.5 | 1.2 ± 0.4 | 1.4 ± 0.6 | 0.899† | 0.412 | NS |

Test values for categorical variables are Chi-square (χ²) values; for continuous variable (†) it is F-value from ANOVA.
VAP: Ventilator-Associated Pneumonia; CVL: Central Venous Line; IJV: Internal Jugular Vein; AKI: Acute Kidney Injury (defined and staged per KDIGO criteria). Values are presented as number (percentage). NS = Non-significant (p > 0.05).

**Supplementary Table (3):** Comparison between the three studied groups regarding mechanical ventilation and type of sedation used

| **Variable** | **Group I (n=30)** | **Group II (n=30)** | **Group III (n=30)** | **Test value*** | **p-value** | **Sig.** |
| --- | --- | --- | --- | --- | --- | --- |
| **MV** |  |  |  |  |  |  |
| **- No** | 6 (20.0%) | 6 (20.0%) | 4 (13.3%) | 0.608 | 0.738 | NS |
| **- Yes** | 24 (80.0%) | 24 (80.0%) | 26 (86.7%) |  |  |  |
| **Propofol** |  |  |  |  |  |  |
| **- No** | 17 (56.7%) | 13 (43.3%) | 11 (36.7%) | 2.509 | 0.285 | NS |
| **- Yes** | 13 (43.3%) | 17 (56.7%) | 19 (63.3%) |  |  |  |
| **Morphine** |  |  |  |  |  |  |
| **- No** | 24 (80.0%) | 24 (80.0%) | 22 (73.3%) | 0.514 | 0.773 | NS |
| **- Yes** | 6 (20.0%) | 6 (20.0%) | 8 (26.7%) |  |  |  |
| **Fentanyl** |  |  |  |  |  |  |
| **- No** | 12 (40.0%) | 12 (40.0%) | 12 (40.0%) | 0.000 | 1.000 | NS |
| **- Yes** | 18 (60.0%) | 18 (60.0%) | 18 (60.0%) |  |  |  |
| **Midazolam** |  |  |  |  |  |  |
| **- No** | 19 (63.3%) | 23 (76.7%) | 23 (76.7%) | 1.772 | 0.412 | NS |
| **- Yes** | 11 (36.7%) | 7 (23.3%) | 7 (23.3%) |  |  |  |

Values are presented as number (percentage).
NS = Non-significant (p > 0.05). MV = Mechanical ventilation.

**Supplementary Table (4):** Comparison between the three studied groups regarding discharge outcomes

|  | | **Group I** | **Group II** | **Group III** | **Test**  **value** | **P-value** | **Sig.** |
| --- | --- | --- | --- | --- | --- | --- | --- |
|  |  | No. = 30 | No. = 30 | No. = 30 |  |  |  |
| **Improvement** | Discharged to home | 20 (66.7%) | 24 (80.0%) | 25 (83.3%) | 2.609 | 0.271 | NS |
|  | Died | 10 (33.3%) | 6 (20.0%) | 5 (16.7%) |  |  |  |
| **Cause of death** | Septic shock | 3 (30.0%) | 2 (33.3%) | 1 (20.0%) | 0.257 | 0.880 | NS |
|  | Brain death | 7 (70.0%) | 4 (66.7%) | 4 (80.0%) |  |  |  |

NS = Non-significant (p > 0.05).

**Supplementary Table (5):** Comparison between the three studied groups regarding GCS among alive patients

| **GCS in Alive Patients** | **Group I (n=20)** | **Group II (n=24)** | **Group III (n=25)** | **Test value** | **P-value** | **Sig.** |
| --- | --- | --- | --- | --- | --- | --- |
| **At admission** | 9.00 ± 2.00 (6–12) | 9.00 ± 2.00 (6–12) | 8.00 ± 2.00 (5–12) | 0.525 | 0.594 | NS |
| **On discharge** | 12.55 ± 2.70 (7–15) | 13.88 ± 1.87 (9–15) | 13.36 ± 2.08 (8–15) | 1.967 | 0.148 | NS |
| **Paired t-test** | 7.384 | 11.942 | 15.325 |  |  |  |
| **P-value (within group)** | <0.001 | <0.001 | <0.001 |  |  | HS |

NS = Non-significant (p > 0.05); HS = Highly significant (p < 0.01); SD = Standard deviation; GCS = Glasgow Coma Scale.

**Supplementary Table (6):** Daily Median Heart Rate (bpm) and Systolic Blood Pressure (mmHg) with Interquartile Range (IQR) by Study Group over the 14-Day Observation Period

| Day | Group I (Control) | Group II (Propranolol) | Group III (Combination) |
| --- | --- | --- | --- |
| **HR (bpm), Median (IQR)** | | | |
| 0 | 115 (113 - 117) | 114 (112 - 116) | 116 (114 - 118) |
| 1 | 118 (116 - 120) | 105 (103 - 107) | 102 (100 - 104) |
| 2 | 125 (122 - 128) | 98 (96 - 100) | 95 (93 - 97) |
| 3 | 122 (119 - 125) | 92 (90 - 94) | 88 (86 - 90) |
| 4 | 130 (127 - 133) | 90 (88 - 92) | 86 (84 - 88) |
| 5 | 128 (125 - 131) | 88 (86 - 90) | 85 (83 - 87) |
| 6 | 120 (117 - 123) | 87 (85 - 89) | 84 (82 - 86) |
| 7 | 118 (115 - 121) | 86 (84 - 88) | 83 (81 - 85) |
| 8 | 124 (121 - 127) | 85 (83 - 87) | 82 (80 - 84) |
| 9 | 119 (116 - 122) | 85 (83 - 87) | 82 (80 - 84) |
| 10 | 116 (113 - 119) | 84 (82 - 86) | 81 (79 - 83) |
| 11 | 113 (110 - 116) | 84 (82 - 86) | 81 (79 - 83) |
| 12 | 112 (109 - 115) | 83 (81 - 85) | 80 (78 - 82) |
| 13 | 111 (108 - 114) | 83 (81 - 85) | 80 (78 - 82) |
| 14 | 110 (107 - 113) | 83 (81 - 85) | 80 (78 - 82) |
| **SBP (mmHg), Median (IQR)** | | | |
| 0 | 134 (130 - 138) | 132 (128 - 136) | 136 (132 - 140) |
| 1 | 142 (138 - 146) | 128 (124 - 132) | 125 (121 - 129) |
| 2 | 148 (144 - 152) | 122 (118 - 126) | 118 (114 - 122) |
| 3 | 145 (141 - 149) | 118 (114 - 122) | 112 (108 - 116) |
| 4 | 152 (148 - 156) | 116 (112 - 120) | 110 (106 - 114) |
| 5 | 150 (146 - 154) | 114 (110 - 118) | 108 (104 - 112) |
| 6 | 142 (138 - 146) | 112 (108 - 116) | 106 (102 - 110) |
| 7 | 140 (136 - 144) | 110 (106 - 114) | 104 (100 - 108) |
| 8 | 146 (142 - 150) | 108 (104 - 112) | 102 (98 - 106) |
| 9 | 141 (137 - 145) | 108 (104 - 112) | 102 (98 - 106) |
| 10 | 138 (134 - 142) | 106 (102 - 110) | 100 (96 - 104) |
| 11 | 135 (131 - 139) | 106 (102 - 110) | 100 (96 - 104) |
| 12 | 134 (130 - 138) | 104 (100 - 108) | 98 (94 - 102) |
| 13 | 133 (129 - 137) | 104 (100 - 108) | 98 (94 - 102) |
| 14 | 132 (128 - 136) | 104 (100 - 108) | 98 (94 - 102) |

Values are presented as median with interquartile range (IQR) for Group I (Control), Group II (Propranolol), and Group III (Combination therapy) from Day 0 (therapy initiation) to Day 14. Both treatment groups (II and III) exhibit a sustained reduction in median values throughout the monitoring period compared to the control group, with Group III showing the most pronounced and stable attenuation. This pattern confirms the expected pharmacological effect of the interventions on key autonomic parameters. Abbreviations: bpm: beats per minute; HR: heart rate; IQR: interquartile range; SBP: systolic blood pressure.
